# A Peptide Found in Human Serum, Derived from the C-Terminus of Albumin, Shows Antifungal Activity In Vitro and In Vivo

**DOI:** 10.3390/microorganisms8101627

**Published:** 2020-10-21

**Authors:** Tecla Ciociola, Pier Paolo Zanello, Tiziana D’Adda, Serena Galati, Stefania Conti, Walter Magliani, Laura Giovati

**Affiliations:** 1 Department of Medicine and Surgery, University of Parma, 43126 Parma, Italy; tecla.ciociola@unipr.it (T.C.); pierpaolo.zanello@libero.it (P.P.Z.); tiziana.dadda@unipr.it (T.D.); walter.magliani@unipr.it (W.M.); laura.giovati@unipr.it (L.G.); 2 Department of Chemistry, Life Sciences and Environmental Sustainability, University of Parma, 43124 Parma, Italy; serena.galati@unipr.it

**Keywords:** antifungal peptides, *Candida albicans*, confocal microscopy, cryptides, electron microscopy, *Galleria mellonella* model, natural antimicrobials

## Abstract

The growing problem of antimicrobial resistance highlights the need for alternative strategies to combat infections. From this perspective, there is a considerable interest in natural molecules obtained from different sources, which are shown to be active against microorganisms, either alone or in association with conventional drugs. In this paper, peptides with the same sequence of fragments, found in human serum, derived from physiological proteins, were evaluated for their antifungal activity. A 13-residue peptide, representing the 597–609 fragment within the albumin C-terminus, was proved to exert a fungicidal activity in vitro against pathogenic yeasts and a therapeutic effect in vivo in the experimental model of candidal infection in *Galleria mellonella*. Studies by confocal microscopy and transmission and scanning electron microscopy demonstrated that the peptide penetrates and accumulates in *Candida albicans* cells, causing gross morphological alterations in cellular structure. These findings add albumin to the group of proteins, which already includes hemoglobin and antibodies, that could give rise to cryptic antimicrobial fragments, and could suggest their role in anti-infective homeostasis. The study of bioactive fragments from serum proteins could open interesting perspectives for the development of new antimicrobial molecules derived by natural sources.

## 1. Introduction

Despite the recent extraordinary advances in medical fields leading to a significant increase in life expectancy, especially in rich and industrialized countries, paradoxically these same progresses have been linked to an increased risk of the emergence or re-emergence of infectious diseases. An ageing population contributes to an increased risk of infections, even in the absence of other co-morbidities. At the same time, the number of people with underlying diseases or conditions that favor the onset of infectious diseases is growing all over the world. In addition, premature or underweight infants, the elderly, transplant recipients, individuals with tumors, infected with HIV, or with impaired or diminished defenses are more frequently affected by severe infections.

Moreover, as stated by the World Health Organization, antimicrobial resistance poses a huge threat to human health. The direct consequences of infection with resistant microorganisms include longer illnesses, increased mortality, prolonged stays in hospital, loss of protection for patients undergoing surgery and other medical procedures, as well as a significant growth in healthcare costs [1]. To cope with the problems posed by the spread of resistant microbial strains and to treat immunocompromised subjects more adequately, it is increasingly urgent to develop new selective and safe therapeutic agents, with mechanisms of action possibly different from those of the antimicrobials currently in use.

In recent years the study of serum proteome gained increasing importance, particularly in the diagnostic field [2,3,4]. Human serum is one of the most informative body fluids in which tens of thousands of peptides of different molecular weights are present. The complexity of serum proteome is increased by proteolytic enzymes responsible for the cleavage of proteins capable of generating smaller peptides. The formation of biologically active peptides from precursor proteins is a widespread phenomenon in nature. An increasing amount of experimental evidence shows that proteolytic cuts of proteins can give rise to peptides “hidden” in the sequence of the original protein and endowed with activities that are different, in whole or in part, from those of the protein from which they derive. These peptides have been termed cryptides, or crypteins, and their study is known as cryptomics [5,6,7,8,9]. A number of cryptides endowed with antimicrobial activity deriving from very common sources, such as mammalian hemoglobin, saliva and milk proteins, have been described [9,10].

On the basis of previous results, demonstrating that synthetic peptides with sequence equal to fragments of the variable and constant regions of antibodies (Ab) could exert in vitro, ex vivo and in vivo antimicrobial, antiviral, and/or immunomodulatory activities [11,12,13,14], liquid chromatography coupled to high-resolution mass spectrometry (LC-HRMS) was applied to human serum samples, with the aim of identifying antimicrobial Ab-fragments. An Ab-fragment potentially cleaved from IgM chain µ, termed K40H, was detected in serum and proved to exert antifungal and antiviral activities [15].

The aim of this work was to investigate whether peptides found in human serum, derived from physiological proteins, could possess antimicrobial activity. A 13-mer peptide (K13L), deriving from the C-terminal region of serum albumin (fragment 597–609, UniProtKB—P02768), proved to exert a fungicidal activity in vitro against *Candida albicans*, including isolates resistant to antifungal drugs currently in use. It also exhibited a significant therapeutic effect in vivo against experimental systemic candidiasis in *Galleria mellonella*. Larvae of the greater wax moth are a common alternative in vivo host for studying fungal pathogens, pharmacological agents and toxins [16,17,18,19,20]. Conversely, K13L did not show toxic or genotoxic effects against mammalian cells in vitro, nor in vivo toxicity in the invertebrate animal model, thus providing a proof of concept of the antimicrobial potential of serum protein fragments, which could represent a novel source of anti-infective agents.

## 2. Materials and Methods

### 2.1. Selection and Synthesis of Peptides

While searching for antimicrobial Ab-fragments in human serum by LC-HRMS [15], several peptides were detected. The presence in the human serum proteome of some of them, which were selected for this study, was already documented [21,22,23,24]. The selected peptides, K13L, S17K, G17K, D15T, and D15R (Table 1), derived from albumin, fragments C3 and C4 of complement, and fibrinogen alpha chain, were synthesized by solid phase peptide synthesis method using a multiple peptide synthesizer (SyroII, MultiSynTech GmbH), at CRIBI Biotechnology Center (University of Padua, Italy), as previously described [15]. The peptides (purity 80–90% by analytical reverse phase HPLC) were solubilized in dimethylsulfoxide (stock solution, 20 mg/mL). Dilutions in sterile distilled water were made for experimental use. Controls (in the absence of peptides) always contained dimethylsulfoxide at the proper concentration.

### 2.2. Evaluation of the In Vitro Candidacidal Activity of Peptides

The fungicidal activity of the selected peptides was evaluated in vitro by a previously described colony forming unit (CFU) assay [15]. All peptides were preliminarily tested at the concentration of 100 μg/mL vs. the reference strain *C. albicans* SC5314. The peptides that exhibited fungicidal activity in the preliminary assay were subsequently tested at scalar dilutions to determine the half maximal effective concentration (EC_50_) values against the selected fungal strains *C. albicans* CA-6, SA40, AIDS68 (fluconazole resistant), and UM4 (caspofungin resistant), and *C. glabrata* OMNI32 (fluconazole, itraconazole, and voriconazole resistant) [13]. After incubation at 37 °C for 6 h in the presence or absence (control growth) of the peptides, fungal cell suspensions (approximately 500 cells/100 μL of distilled water) were spread on Sabouraud Dextrose agar plates. After incubation at 30 °C for 48–72 h, CFUs were enumerated. Each assay was carried out in triplicate and at least 2 independent experiments were performed for each condition. EC_50_ values were calculated by nonlinear regression analysis using Graph Pad Prism 4.01 software, San Diego, CA, USA. Time kinetics of K13L-mediated killing against the reference *C. albicans* strain was determined by CFU assays, as described above, after incubation of yeast cells for 30 min, 1, 2, 4 and 6 h with K13L at different concentrations.

### 2.3. Evaluation of the Hemolytic, Cytotoxic, and Genotoxic Activity of Selected Peptides

The hemolytic and cytotoxic activity of the selected peptides (25, 50 and 100 μM) was evaluated as previously described [15]. For the evaluation of hemolytic activity, peptides were added to human erythrocytes (blood group 0 Rh+, final concentration, 2.5% *v*/*v*) and hemoglobin release was estimated after 30 and 120 min incubation by measuring the absorbance of the supernatant at 540 nm. Erythrocytes suspended in phosphate buffered saline (PBS) and Triton 1% were the controls for zero (blank) and 100% hemolysis, respectively. Cytotoxicity against Rhesus monkey kidney epithelial cells (LLC-MK2 line) treated with peptides for 24 h was assessed after resazurin addition by measuring fluorescence intensity at 572 nm. Cells in medium without peptide served as control (100% viability). Each assay was carried out in duplicate and at least 2 independent replicates were performed for each experiment.

According to a previously described procedure [15], the alkaline Comet assay was used to evaluate the genotoxic activity of selected peptides. Human peripheral blood mononuclear cells (PBMCs) were treated for 120 min with the peptides (20 μM) that proved to be active against the reference *C. albicans* SC5314 strain. Data were collected by an automatic image analysis system (Comet Assay III, Perceptive Instruments Ltd.) after examination with a fluorescent microscope (Leica DMLS). DNA migration was evaluated by percentage of DNA in comet tail (Tail Intensity, expressed as mean values). The assay was carried out in duplicate.

Data are reported as the mean ± SD. Statistical significance was assessed by *t* test, *p* < 0.05 was considered significant.

### 2.4. Circular Dichroism (CD) Spectroscopy

Far-UV CD spectra (range 190–250 nm) were acquired with a Jasco J715 Spectropolarimeter coupled to a Peltier temperature controller, using a 1 mm path-length cuvette, a bandwidth of 1 nm, a data pitch of 0.5 nm, and a response time of 4 s; CD spectra were averaged from 4 scans at 20 °C. K13L samples (final concentration 100 μM) were analyzed immediately or 5 days after preparation of the starting aqueous solution (1.58 mM). CD spectra of K13L (50 μM) were acquired also in presence of 100 mM sodium dodecyl sulfate (SDS). Following baseline correction, the measured ellipticity, θ (mdeg), was converted to the molar mean residue ellipticity [θ] (deg·cm^2^·dmol^−1^).

### 2.5. Evaluation of Apoptosis Induction in C. albicans Cells after Treatment with K13L

The Muse cell analyzer (Merck Millipore, Germany) was used to evaluate peptide-induced apoptosis in *C. albicans* SC5314 cells, as previously described [25]. Germinating yeast cells (5 × 10^5^ cells/mL) were incubated for 30 min at room temperature in the absence (control) or presence of K13L at a concentration equal to 2 × EC_50_. After addition of the Muse^®^ Annexin V and Dead Cell reagent, treated and control cell suspensions were maintained for 20 min in the dark before data acquisition, according to the manufacturer’s instructions. Two independent experiments were performed, each carried out in duplicate. A CFU assay was performed to verify the candidacidal activity of the peptide under the adopted conditions.

### 2.6. Evaluation of In Vivo Toxicity and Therapeutic Activity of Peptide

The potential therapeutic activity of K13L was evaluated by the *G. mellonella* model of experimental candidiasis, according to a previously described procedure [15,25]. To preliminarily evaluate in vivo toxicity, a solution of K13L (33.33 mg/kg) was inoculated directly into the haemocoel, via the last left pro-leg, in larvae at their final instar stage (body weight, 300 ± 20 mg, 10 μL/larva). Larvae inoculated with 10 μL of saline solution and untouched larvae served as controls. All animals were incubated at 37 °C in the dark and monitored daily for survival for 9 days. For the evaluation of therapeutic activity, larvae (16/group) were challenged with *C. albicans* SC5314 (5 × 10^5^ yeast cells/larva, 10 μL) via the last left pro-leg. A solution of K13L (16.66 mg/kg,) or saline (control) was injected 60 min later, via the last right proleg (10 μL). Larvae were monitored daily for 9 days after infection. The log rank (Mantel–Cox) test was used to compare survival curves by Graph Pad Prism software.

### 2.7. Transmission and Scanning Electron Microscopy Studies

Transmission and scanning electron microscopy (TEM and SEM) studies on K13L-treated *C. albicans* SC5314 cells were performed as previously described [25]. For TEM, yeast cells (7.5 × 10^6^ in 50 μL) were incubated for 60 min with K13L (250 μg/mL), then pre-fixed with 5% glutaraldehyde, packed in solidified 3% agarose, fixed for 3 h at room temperature with 2.5% glutaraldehyde and kept overnight at 4 °C. After post-fixation with 1% osmium tetroxide (30 min) and dehydration with acetone gradient (25–100%), Durcupan ACM epoxy resin was used for infiltration, followed by embedding and hardening of the resin for 72 h at 58 °C. Ultra-thin sections (80 nm) were contrasted with 4% uranyl acetate and Reynolds’ lead citrate prior examination with a Philips EM 208S transmission electron microscope (Fei Europe, Eindhoven, The Netherlands).

For SEM studies, yeast cells (4 × 10^5^ in 20 μL) were incubated with K13L (125 μg/mL) for 60 min, then placed on 25-mm^2^ glass slides. After fixation with a glutaraldehyde-sodium cacodylate buffer and washing in sodium cacodylate, dehydration was obtained by alcohol gradient (25–100%). Samples washed in acetone were dried in liquid CO_2_, fixed on a support and gold coated in an ion-sputtering unit, then observed in a Philips 501 scanning electron microscope (15 kV).

A CFU assay was performed to verify the candidacidal activity of the peptide under the adopted conditions.

### 2.8. Confocal Microscopy Studies

Confocal microscopy studies were performed on living yeast cells according to a procedure previously described [25]. A suspension of *C. albicans* SC5314 cells (4 × 10^5^ in 20 μL) were seeded on a coverslip mounted in a special flow chamber. After 30 min, fluorescein isothiocyanate (FITC)-labeled K13L was added (final concentration 180 μg/mL) and images were taken up to 390 min. Propidium iodide (PI) was finally added (1.5 μM). As described above, a CFU assay was performed to verify the candidacidal activity of the peptide under the adopted conditions.

## 3. Results

### 3.1. In Vitro Fungicidal Activity of Selected Peptides

**Figure 1:**
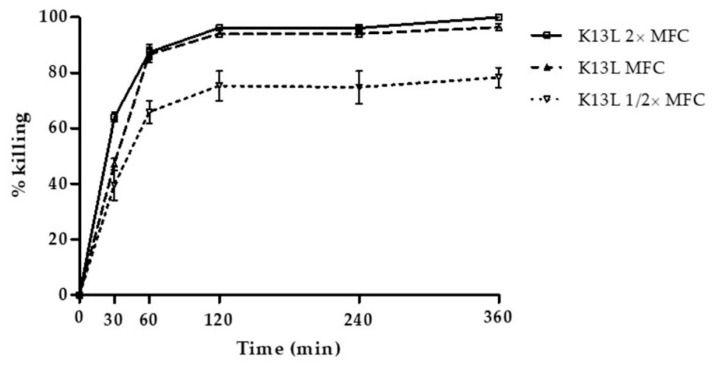
Time kinetics of K13L killing of *C. albicans* SC5314 cells. The activity is expressed as percentage killing, calculated as: 100-(average number of colony forming units (CFUs) in the peptide-treated group/average number of CFUs in the control group) × 100. Each experiment was performed in triplicate.

In preliminary assays performed against the reference *C. albicans* SC5314 strain at the concentration of 100 μg/mL, peptides S17K, D15T, and D15R showed no activity. The activity of peptides K13L and G17K, that proved to be active against the reference strain, was evaluated against all the selected candidal strains and the obtained EC_50_ values are reported in Table 2. Time-killing curves, determined by incubation over time of *C. albicans* SC5314 cells with the most active peptide, K13L, at 1/2, 1, and 2× its minimal fungicidal concentration (MFC), are shown in Figure 1. The curves presented a similar trend at all concentrations used. In particular, killing activity at the MFC value was about 50%, 81%, and 94% after 30, 60, and 120 min of incubation, respectively.

### 3.2. Hemolytic, Cytotoxic, and Genotoxic Effects of Selected Peptides

The selected peptides did not show a significant hemolytic activity, either after 30 or 120 min of incubation (Table 3). The mean absorbance values of released hemoglobin did not differ significantly from the ones of the negative control (PBS) and less than 2% of the erythrocytes lysed in comparison to the negative control (PBS, 0% lysis) even at the highest tested concentration (100 μM). Moreover, the selected peptides were not cytotoxic against LLC-MK2 cells. In fact, at all the tested concentrations, the mean values of fluorescence intensity obtained in the resazurin test were not significantly different from the ones of control cells in the absence of peptides. In Table 4, results of the cytotoxicity assay are expressed as cell viability (%) in comparison to control (in the absence of peptides, 100% viability).

The genotoxic activity of the most active peptides (K13L and G17K) was evaluated by the Comet assay on PBMCs. Tail intensity values for PBMCs treated with 5, 10, and 20 μM K13L and G17K (Table 5) were very low (always <1%, a value that cannot be considered representative of DNA damage), as were those detected in control cells.

### 3.3. K13L Conformational State

**Figure 2:**
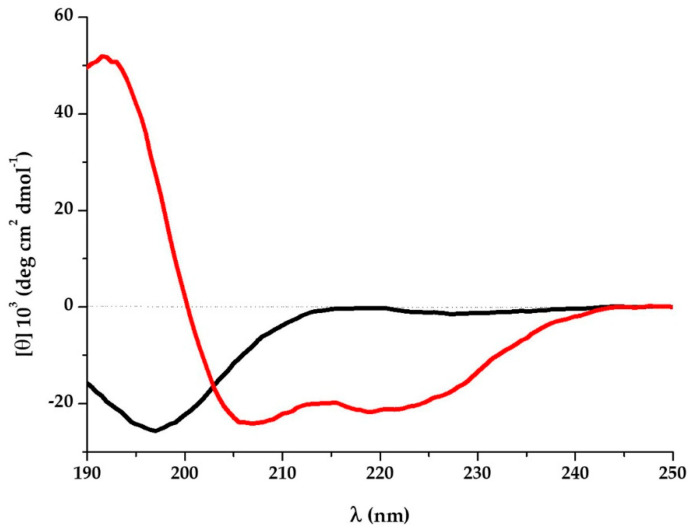
Far-UV circular dichroism (CD) spectra of K13L (100 μM) in aqueous solution (black line) or K13L (50 μM) in 100 mM sodium dodecyl sulfate (SDS) (red line) 5 days after preparation of the starting aqueous solution (1.58 mM).

K13L in aqueous solution showed a typical random coil conformation, with a negative band at 197 nm. In the presence of SDS micelles the peptide acquired a well-defined α-helix, with negative bands at 219 nm and 207 nm and a positive band at 192 nm (Figure 2). The conformation attained by K13L in a hydrophobic environment reflects the α-helix propensity of the primary sequence and is in accordance with its structure inside the human serum albumin (PDB 1BJ5).

### 3.4. Induction of Apoptosis in C. albicans Cells by Treatment with K13L

Induction of apoptosis in *C. albicans* SC5314 whole cells treated with K13L was evaluated by phosphatidylserine externalization and reactivity with annexin V through flow cytometry. After 30 min incubation with the peptide at 2 × EC_50_, the percentage of apoptotic cells on the total gated cells was 0.35 ± 0.14%, not significantly different, as assessed by *t* test, in comparison with untreated control cells (0.35 ± 0.26%). Results of one representative experiment are shown in Supplementary Materials Figure S1. The candidacidal activity of the peptides under the adopted experimental conditions was confirmed by the CFU assay.

### 3.5. In Vivo Toxicity and Therapeutic Activity of K13L

**Figure 3:**
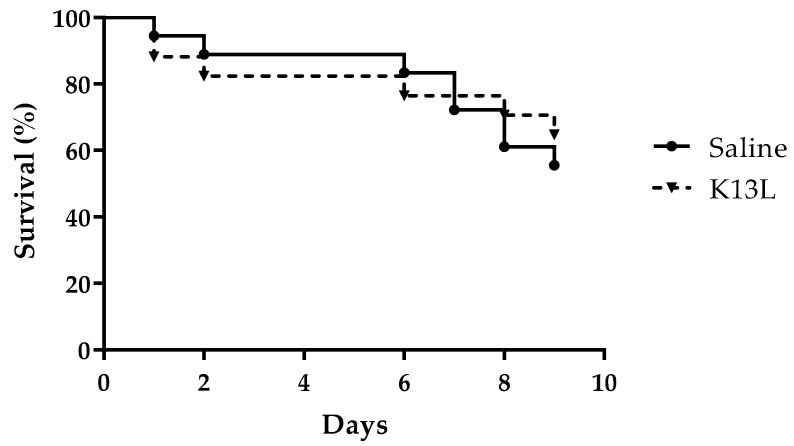
In vivo toxicity of K13L. ●, saline-injected larvae; ▼, K13L-injected larvae. No significant difference in survival was found by the Mantel–Cox log-rank test.

**Figure 4:**
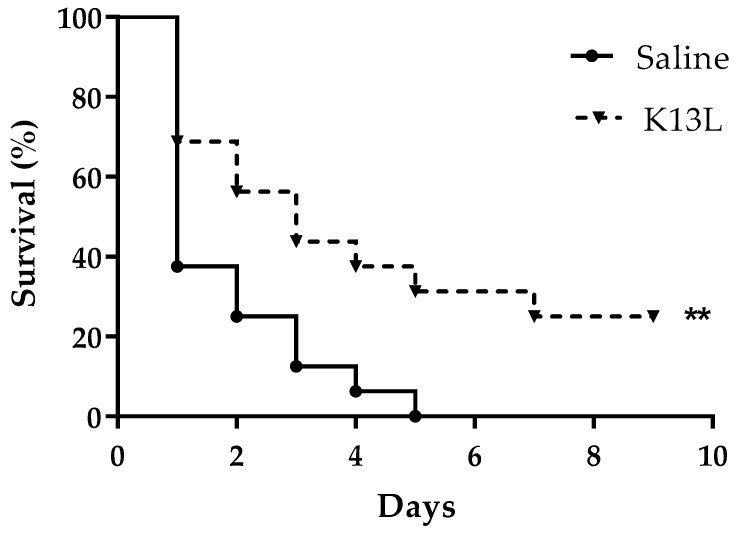
In vivo therapeutic activity of K13L. ●, *C. albicans*-infected, saline-injected larvae; ▼, *C. albicans*-infected, K13L-treated larvae. ** significant difference in survival in comparison to the infected, saline-treated group (*p* = 0.0098), as assessed by the Mantel–Cox log-rank test.

The *G. mellonella* model was adopted to evaluate peptide toxicity to the host and therapeutic activity against *C. albicans* infection. No significant difference in survival was detected between larvae inoculated with K13L (33.33 mg/kg) in comparison to those inoculated with saline (control group), thus showing the lack of toxicity of the peptide in this model (Figure 3). In two independent experiments, a single administration of K13L (16.66 mg/kg) 60 min after candidal infection led to a significant increase in larvae survival in comparison to that of the control group, i.e., infected larvae injected with saline. The survival curves of one representative experiment are shown in Figure 4. Median survival time was 72 h in K13L-treated group vs. 24 h in saline-injected control group. While 100% of the untreated larvae were dead 5 days post-infection, 4/16 of the K13L-treated larvae were still alive at day 9.

### 3.6. Visualization of the Effects of K13L Treatment on C. albicans Cells by Transmission and Scanning Electron Microscopy

**Figure 5:**
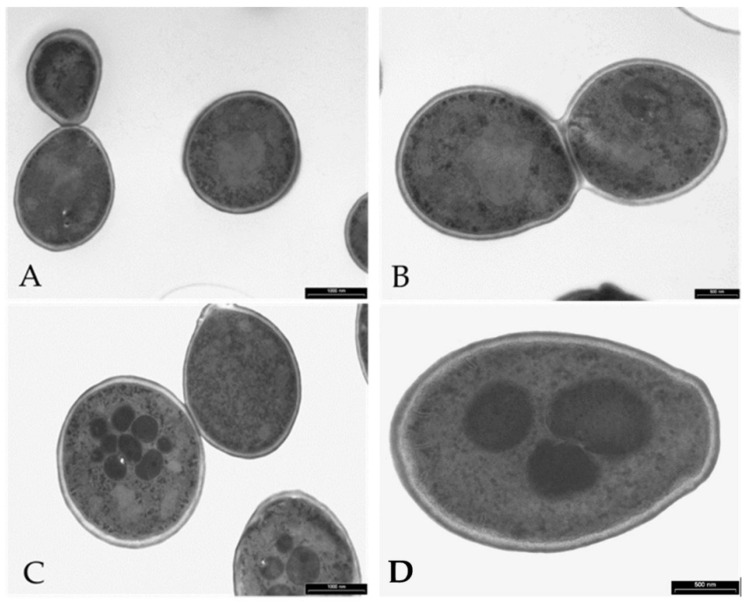
Transmission electron microscopy of *Candida albicans* cells treated with peptide K13L. Cells were incubated without (panels **A** and **B**) or with (panels **C** and **D**) K13L. Microbodies were seen in still intact treated cells. Bar: 1000 nm (panels **A** and **C**), 500 nm (panels **B** and **D**).

**Figure 6:**
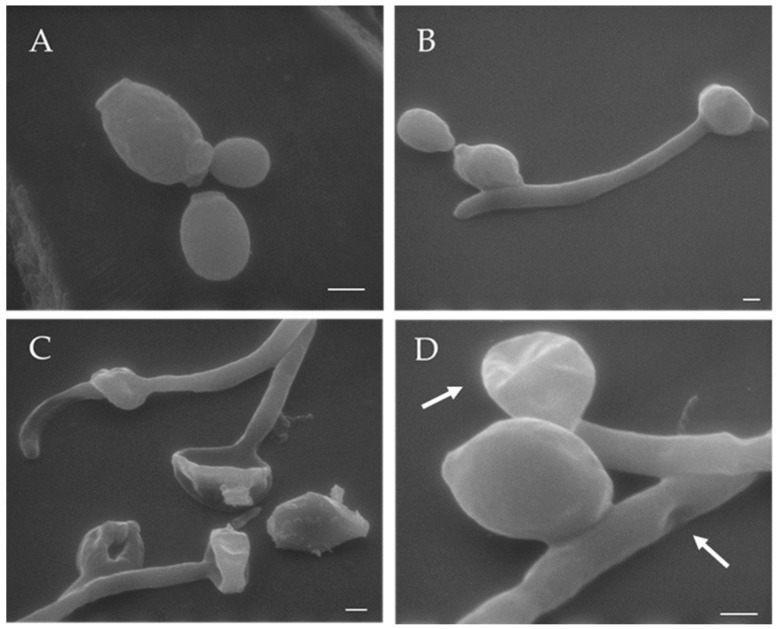
Scanning electron microscopy of *Candida albicans* cells untreated (control, panels **A** and **B**) or treated with peptide K13L (panels **C** and **D**). Gross alterations in cell morphology were observed after treatment with the peptide (panel **C** and panel **D**, arrows). Bar: 1 μm.

In comparison to untreated controls, treatment with K13L caused alterations in the morphology of *C. albicans* cells (Figure 5 and Figure 6). In transmission electron microscopy specimens, intracellular structures of variable dimensions, which can be defined as “microbodies” (peroxisomes), were observed in still intact yeast cells after K13L treatment. Scanning electron microscopy showed most yeast cells visibly damaged, with gross morphological alterations.

### 3.7. Peptide–Yeast Cells Interaction

**Figure 7:**
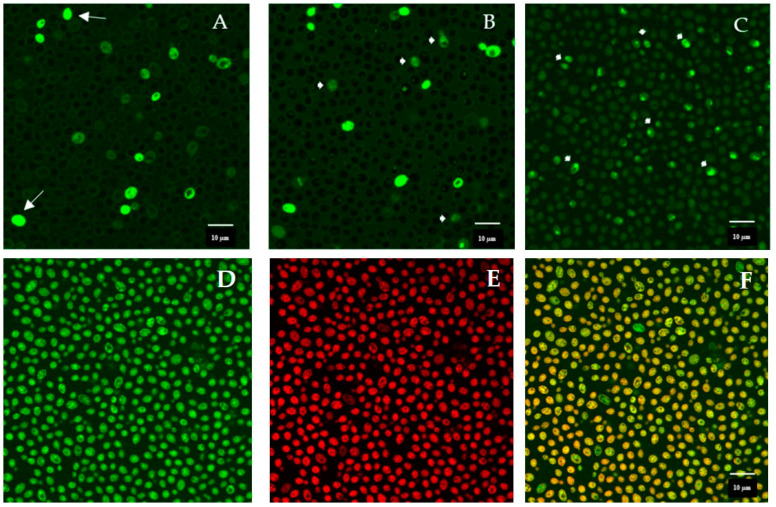
Confocal microscopy images of *Candida albicans* cells treated with fluorescein isothiocyanate (FITC)-labeled K13L for 5 min (panel **A**), 75 min (panel **B**), 285 min (panel **C**), and 315 min, 30 min after addition of propidium iodide (PI) (panel **D**, FITC, panel **E**, PI, panel **F**, merge). Some non-viable yeast cells are already present in the inoculum (panel **A**, arrows); over time, intracellular localization of FITC-labeled K13L is observed (some cells indicated by arrowheads, panels **B** and **C**). Peptide internalization led to cell death, as demonstrated by the merge of green (FITC) and red (PI) signals in panel **F**).

Cells of *C. albicans* SC5314 treated with FITC-labeled K13L peptide at the concentration of 180 µg/mL observed at different time intervals are shown in Figure 7. Immediately after the addition of K13L, non-viable yeast cells already present in the inoculum showed a diffused fluorescence in the cytoplasm, while, over time, the peptide entered most viable yeast cells with a progressive increase in intracellular localization. The addition of PI to the inoculum made it possible to observe cell death induced by peptide internalization.

## 4. Discussion

It is known that fragments of physiological proteins, possibly released in vivo through proteolytic cleavage, could represent functional units characterized by biological activities not predictable from the sequence or the activity of the original protein [9]. This study is focused on the antifungal activity of peptide fragments derived from proteolytic degradation of serum proteins and found in vivo in human serum. The albumin-derived peptide K13L showed a fungicidal activity against *C. albicans*, including strains resistant to conventional antifungal drugs, at micromolar concentration. K13L, in comparison to the other investigated peptides characterized by a lower (G17K) or absent candidacidal activity, presents a positive grand average of hydropathy (GRAVY) value and a net positive charge, a feature shared by many antimicrobial peptides. It is widely recognized that a net positive charge and a characteristic alternation in the sequence of hydrophilic/hydrophobic amino acids is of fundamental importance for the interaction with the negatively charged microbial surface and for the activity of antimicrobial peptides, regardless of their mechanism of action. Likewise K13L, the naturally occurring Ab-fragment K40H presents a net positive charge, as well as other peptides derived from Ab constant regions (N10K and T11F) or encoded by immunoglobulin genes (L12P and L18R) described in our previous papers [13,25]. It has also been demonstrated that the replacement of positively charged residues with alanine causes a reduction in candidacidal activity [13]. CD spectroscopy analysis revealed a random coil conformation of K13L in aqueous solution and the conversion to α-helix structure after the addition of SDS (membrane mimetic environment). A large number of natural antimicrobial peptides adopt a α-helical conformation for binding and disruption of microbial membranes. However, the time-killing curve of K13L suggests that candidacidal activity is not related to a pore-forming mode of action. In fact, in comparison to other investigated fungicidal peptides, i.e., the previously described L12P and L18R peptides, the killing effect was slower. Likewise, confocal microscopy studies with FITC-labeled K13L showed a progressive internalization of the peptide over time, leading to cell death after 4 h, while L12P and L18R were rapidly internalized and most yeast cells were no longer viable after 60 min [25]. The α-helical conformation could allow K13L binding and lead to an increase in membrane permeability enabling peptide penetration inside the cell where one or more intracellular targets could be involved in its mode of action, as previously described for other antimicrobial peptides [26]. While no apoptotic activity has been observed after K13L treatment, images obtained by transmission electron microscopy showed in several treated and still intact *C. albicans* cells the formation of “microbodies”, described in yeast cells also following oxidative stress as the site of the main enzymatic processes catalyzed by deoxidative enzymes. Even if no detectable quantities of intracellular oxygen free radicals were observed following treatment with K13L (data not shown), it could be that the action of the peptide may generate precursors that have a role in microbody formation. Eventually, peptide treatment led to gross alterations in cellular structure as evidenced by scanning electron microscopy images. Although further studies are needed to elucidate the exact mechanism of K13L candidacidal activity, the peptide was proved to exert a significant therapeutic activity in vivo in the *G. mellonella* model of systemic candidiasis.

The demonstration of the antimicrobial activity of K13L allows the inclusion of albumin in the large group of proteins from which type 1 cryptids can be derived, according to the definition of Autelitano et al. [5]. These cryptides increase the spectrum of biological activities associated with a particular protein, offering interesting prospects in the field of therapies based on the use of peptides [27]. Antimicrobial peptides, in particular, have already been suggested in the past few decades as new anti-infective therapeutic agents [28,29,30]. The drawbacks related to the use of these molecules in therapeutics can now be overcome, given advances in biotechnology, genetic engineering, and delivery strategies [31,32].

Therefore, antimicrobial peptides such as K13L described in this study could open interesting perspectives, proposing serum precursor proteins as a possible source of leading molecules with anti-infective potential.

## Figures and Tables

**Table 1:** Characteristics of selected peptides.

Peptide	Aminoacidic Sequence	Parent Protein	GRAVY Value ^1^	pI	M.M. (Da) ^2^	Charge ^3^
K13L	KKLVAASQAALGL	Serum albumin	0.792	10.00	1269.53	2+
G17K	GLEEELQFSLGSKINVK	Complement C4-A	−0.282	4.79	1891.13	1−
S17K	SEETKENEGFTVTAEGK	Complement C3	−1.394	4.32	1855.81	3−
D15R	DSGEGDFLAEGGGVR	Fibrinogen α chain	−0.580	3.92	1465.48	3−
D15T	DEAGSEADHEGTHST	Fibrinogen α chain	−1.607	4.17	1542.43	3−

^1^ Grand average of hydropathy (GRAVY) calculated by the ExPASy tool ProtParam; ^2^ Molecular Mass (Dalton); ^3^ Net charge, +: positive, −: negative.

**Table 2:** In vitro fungicidal activity of K13L and G17K against candidal strains.

Fungal Strain	EC50 ^1^ (95% Confidence Intervals) mol/liter × 10^−6^	
	K13L	G17K
*Candida albicans* SC5314	4.29 (3.21–5.76)	18.46 (10.08–33.78)
*C. albicans* CA-6	6.28 (5.09–7.73)	33.74 (22.80–49.93)
*C. albicans* SA40	3.08 (2.50–3.82)	24.78 (19.64–31.26)
*C. albicans* AIDS68	2.48 (2.01–3.05)	28.17 (25.15–31.56)
*C. albicans* UM4	7.43 (4.39–12.56)	23.09 (9.28–57.42)
*C. glabrata* OMNI32	4.45 (4.15–4.76)	13.95 (19.93–15.04)

^1^ half maximal effective concentration, expressed in mol/liter × 10^−6^. In parentheses, 95% confidence intervals.

**Table 3:** In vitro hemolytic activity of the investigated peptides.

Peptide	Hemolysis (%)					
	30 min			120 min		
	25 µM	50 µM	100 µM	25 µM	50 µM	100 µM
K13L	0.29	0.32	0.61	0.00	0.00	1.09 *
G17K	0.00	0.03	0.13	0.00	0.00	0.00
S17K	0.00	0.35	0.80	0.00	0.26	1.12 *
D15R	0.27	0.51	0.85	0.00	0.00	0.38
D15T	0.00	0.00	0.67	0.00	0.00	0.24

SD < 0.01, * *p* < 0.05.

**Table 4:** In vitro cytotoxic activity of the investigated peptides against LLC-MK2 cells.

Peptide	Cell Viability (%)		
	25 µM	50 µM	100 µM
K13L	105.95 ± 1.47	105.04 ± 0.20	101.26 ± 0.38
G17K	99.07 ± 1.57	99.73 ± 0.45	99.07 ± 1.57
S17K	103.47 ± 2.00	102.69 ± 1.37	101.73 ± 1.82
D15R	101.32 ± 1.92	101.25 ± 0.19	103.00 ± 1.49
D15T	108.50 ± 0.92	107.55 ± 1.48	108.50 ± 5.15

**Table 5:** In vitro genotoxic activity of selected peptides.

Peptide	Tail Intensity (%)			
	0	5 µM	10 µM	20 µM
K13L	0.37 ± 0.04	0.69 ± 0.03 *	0.59 ± 0.09 *	0.36 ± 0.22
G17K	0.37 ± 0.04	0.40 ± 0.02	0.34 ± 0.25	0.12 ± 0.05

* *p* < 0.05.

## Supplementary Materials

The following are available online at https://www.mdpi.com/2076-2607/8/10/1627/s1, Figure S1: Apoptotic profile from a single assay performed on *Candida albicans* cells.
