# Supplementary material for: A Peptide Found in Human Serum, Derived from the C-Terminus of Albumin, Shows Antifungal Activity In Vitro and In Vivo
Source: Microorganisms. 2020 Oct 21;8(10):1627. doi: 10.3390/microorganisms8101627 (PMC7588913; doi:10.3390/microorganisms8101627)
Supplement: Supplementary file 1 [file microorganisms-08-01627-s001.pdf]

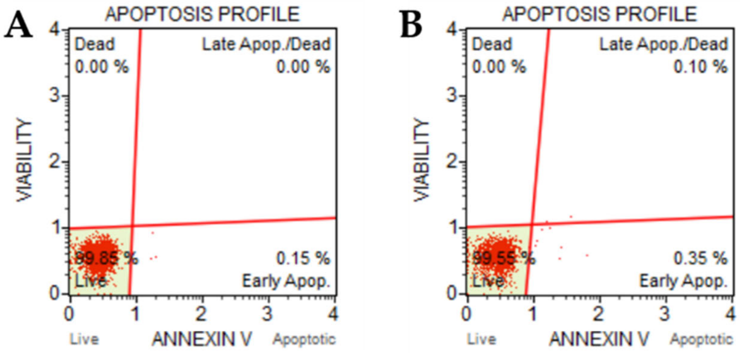

Cell Conc. (Cells / mL)	% Gated		Cell Conc. (Cells / mL)	% Gated
4.26E+05	99.85 %	Live (LL) :	4.34E+05	99.55 %
6.40E+02	0.15 %	Early Apoptotic (LR) :	1.52E+03	0.35 %
0.00E+00	0.00 %	Late Apop./ Dead (UR)	4.36E+02	0.10 %
0.00E+00	0.00 %	Debris (UL) :	0.00E+00	0.00 %
6.40E+02	0.15 %	Total Apoptotic :	1.96E+03	0.45 %

Figure S1. Apoptotic profile from a single assay performed on *Candida albicans* cells. Panel A: untreated cells (control), Panel B: cells treated with K13L for 30 minutes. Non apoptotic dead cells are not included in the number of gated cells, being below the threshold settings based on the size index. LL: lower left quadrant; LR: lower right quadrant; UR: upper right quadrant; UL: upper left quadrant.
